# Extremely severe hypochloremic metabolic alkalosis after ileorectal anastomosis in a patient with chronic intestinal pseudo-obstruction

**DOI:** 10.1093/gastro/goad037

**Published:** 2023-06-30

**Authors:** Peter Heinz-Erian, Andreas R Janecke, Thomas Müller, Peter Rehder, Elisabeth Bruder, Thomas Menter, Heinz Zoller, Markus Pirklbauer, Michael Rieger

**Affiliations:** Department of Pediatric and Adolescent Medicine, Medical University of Innsbruck, Innsbruck, Tyrol, Austria; Department of Pediatric and Adolescent Medicine, Medical University of Innsbruck, Innsbruck, Tyrol, Austria; Department of Pediatric and Adolescent Medicine, Medical University of Innsbruck, Innsbruck, Tyrol, Austria; Department of Urology, Medical University of Innsbruck, Innsbruck, Tyrol, Austria; Institute of Pathology, University Hospital Basel, Basel, Basel, Switzerland; Institute of Pathology, University Hospital Basel, Basel, Basel, Switzerland; Department of Internal Medicine I, Medical University of Innsbruck, Innsbruck, Tyrol, Austria; Department of Internal Medicine IV, Medical University of Innsbruck, Innsbruck, Tyrol, Austria; Department of Radiology, District Hospital Hall, Hall, Tyrol, Austria

## Introduction

Chronic intestinal pseudo-obstruction (CIPO), defined as chronic constipation without mechanical obstruction of the gastrointestinal tract [1, 2], often presents with a truly chameleon-like symptomatology including irregular defecation, abdominal pain and distension, nausea, vomiting, and sometimes fluid/electrolyte imbalance. Although diarrhea was found in ≤17% of CIPO cases, its nature, however, has not been precisely described. Serious deviations of electrolyte and acid–base status, to the best of our knowledge, have not been reported.

Transient fluid/electrolyte imbalances have been described after colectomy [3] but gross failure of the body's ability to maintain homeostasis has not been reported. We here describe a patient with a congenital myopathic form of CIPO who had undergone total resection of the colon because of intractable constipation and who developed severe watery diarrhea and hypochloremic metabolic alkalosis.

## Case presentation

The patient was born (birth weight 3,850 g) as the second child of fourth-degree Caucasian cousins. From the age of 2 months, he was treated for recurrent episodes of constipation that became increasingly enema-dependent. Hirschsprung's disease was excluded at 9 months by ruling out abnormal acetylcholinesterase staining in rectal suction biopsies. At 11 months of age, the patient's left hemicolon was resected for toxic megacolon, followed by resection of the ascending colon, cecum, and ileocecal valve, and the creation of an ileorectal anastomosis at 2 years of age. Unfortunately, no histology reports are available about these surgeries. However, the patient continued to suffer from chronic constipation that was repeatedly interrupted by episodes of severe watery diarrhea that became the clearly prevailing problem with ≤8 L of stool daily.

**Figure 1.**
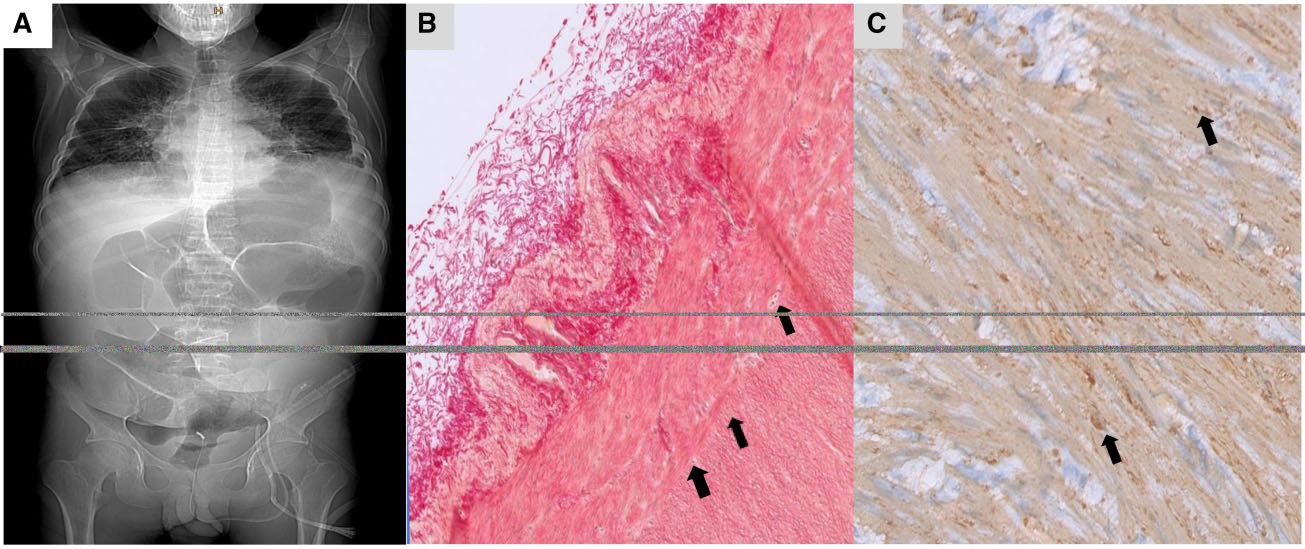
Radiologic and histologic findings characteristic of the patient's diagnosis of megacystis microcolon intestinal hypoperistalsis syndrome. (A) Computed tomography scout of the patient's abdomen showing massively dilated bowel loops. (B) Full-thickness biopsy from the patient's rectum showing severe disarrangement of the rectal wall architecture with a loss of the connective tissue plexus layer and hypoganglionosis (arrows; Sirius red staining, ×150). The uppermost arrow indicates a small ganglion. (C) Granular irregularities in *ACTG2*-expressing smooth muscle cells of the tunica muscularis propria (arrows, smooth muscle actin staining, ×400).

At the age of 17 years, the patient was growth-retarded (157 cm, standard deviation score [SDS] –2.6) and severely underweight (36 kg, SDS –5.5). His abdomen was bloated and painful. Radiographic examinations showed massively dilated bowel loops in the computed tomography scout (Figure 1A) with air–fluid levels on contrast radiography and little peristalsis. No obstructing mass lesion was demonstrated on endoscopy. Jejunal biopsies showed an only slightly reduced villous height but no structural abnormalities of the epithelium by light and electron microscopy. Celiac disease serology under a gluten-containing diet was repeatedly negative. Glucose breath tests were positive on several occasions indicating small bowel bacterial overgrowth (SBBO). Repeated plasma chloride concentrations were as low as 64 mmol/L, as were those of sodium (120 mmol/L) and potassium (2.6 mmol/L), together with extreme metabolic alkalosis (plasma pH 7.78, plasma bicarbonate +58 mmol/L). Fecal chloride concentrations were between 108 and 158 mmol/kg.

In addition to his gastrointestinal symptoms, voiding problems increased with progressively larger volumes of residual urine. Video-urodynamic studies confirmed a large-capacity bladder with an acontractile detrusor.

The severe watery diarrhea together with extremely low plasma chloride concentrations, severe metabolic alkalosis, and large fecal chloride losses now strongly argued for a diagnosis of congenital chloride diarrhea (CCD) [4]. Large amounts of fluids, NaCl and KCl, normalized the patient's hydration and electrolyte status, and markedly improved his overall well-being. He showed remarkable catch-up growth reaching normal height (172 cm, SDS –0.6) and weight (56 kg, SDS –1.3) until his 19th birthday. Although this obvious improvement after adequate fluid–electrolyte supplement further supported the suspicion of CCD, no mutation in *SLC26A3* was identifiable, thus ruling out this diagnosis [5].

Ultimately, while no epithelial fluid–electrolyte transport defect or metabolic–endocrine disorder causing hypochloremic metabolic alkalosis could be verified, genetic analysis identified a dominant *de novo* heterozygous NM_001615.3: c.77G>A p.(Arg257His)-mutation in the *ACTG2* gene on chromosome 2p13.1 [6, 7]. In support of this finding, full-thickness rectal biopsies showed a severe disarrangement of the rectal wall architecture with relative hypoganglionosis (Figure 1B), reduction of Cajal cells, and granular irregularities in *ACTG2*-expressing smooth muscle (Figure 1C). Together with similar findings in biopsies of the extremely dilated bladder [8], this confirmed a diagnosis of megacystis microcolon intestinal hypoperistalsis syndrome (MMIHS).

At the age of 25 years, following a 6-year period of independence from parenteral nutrition, the patient started to suffer from increasingly frequent urinary tract infections with multiresistant Klebsiella, promoted by urine stasis due to MMIHS. Impaired transit of urine was associated with hydronephrosis, nephrocalcinosis, renal insufficiency, and dependence on renal dialysis from the age of 28 years onwards. While the patient was investigated for combined intestinal and renal transplantation, he died shortly after his 30th birthday during another episode of multiresistant Klebsiella urosepsis in his hometown hospital.

## Discussion

This case of MMIHS with extremely severe hypochloremic alkalosis and watery diarrhea is another example of the many manifestations of CIPO. The course of the disease was complicated by resection of the colon and ileocecal valve. In addition, the patient had frequent episodes of SBBO that may have been a consequence of both intestinal hypoperistalsis and loss of the ileocecal valve. The erroneous initial diagnosis of CCD caused by non-consideration of the fact that the rectal stool obtained for chloride concentration determination was actually ileal chyme following the creation of an ileorectal anastomosis containing high fecal chloride concentrations that together with hypochloremic alkalosis simulated a typical CCD-like picture. While usually diarrhea is associated with metabolic acidosis due to the loss of sodium bicarbonate [9], CCD is known to cause metabolic alkalosis because of deficient epithelial chloride/bicarbonate exchange [4] and thus bicarbonate retention. Our patient lost fluid and chloride because his colon had been removed and he therefore suffered from severe hypochloremia and hypovolemia, both also contributing factors to his metabolic alkalosis. Additional factors aggravating diarrhea and chloride losses may have been SBBO, facilitated by the absence of the ileocecal valve, and states of enhanced jejunal secretion as reported previously in a few cases of CIPO [10].

Finally, the results of the genetic analysis providing the diagnosis of an *ACTG2* gene-associated MMIHS explained the patient's earlier history of constipation. This was also supported by the pathohistology findings of full-thickness rectal and bladder-neck biopsies [8].

Our patient’s story illustrates the tremendous problems when trying to correctly diagnose and manage a rare genetic disorder such as MMIHS [6, 7]. Although diarrhea has been described in other patients with MMIHS [10], it has hitherto not been found to cause such a severe CCD-like picture as in our case. Whether colonic resection causing loss of chloride and water, together with enhanced jejunal secretion and SBBO—all resulting in watery diarrhea and volume depletion—may be associated with our patient's extreme hypochloremic alkalosis remains unclear.

## Authors’ Contributions

P.H.E. prepared the conceptualization of the case report; contributed to data collection; and drafted, wrote, and submitted the manuscript; A.R.J. provided and interpreted the genetic data; P.R. was responsible for specific urology data and management; E.B. and T. Menter provided pathology investigations and interpretation; T. Müller and H.Z. contributed gastroenterological data and input; M.P. was responsible for nephrology management and dialysis; M.R. provided and interpreted imaging information. All authors have read and approved the final version of the manuscript.
